# Accuracy measures of 1.5-tesla MRI for the diagnosis of ACL, meniscus and articular knee cartilage damage and characteristics of false negative lesions: a level III prognostic study

**DOI:** 10.1186/s12891-021-04011-3

**Published:** 2021-01-29

**Authors:** Jonathan E. J. Koch, Ron Ben-Elyahu, Basel Khateeb, Michael Ringart, Meir Nyska, Nissim Ohana, Gideon Mann, Iftach Hetsroni

**Affiliations:** 1 Department of Orthopedic Surgery, Meir General Hospital, Kfar Saba, and Sackler Faculty of Medicine, Tel Aviv University, Tsharnichovski street 59, 44281 Kfar Saba, Israel; 2 MOR-MAR Ltd., Hassadna street 6, Kfar Saba, Israel

**Keywords:** 5-tesla, False-negative MRI, Ramp lesion, Meniscocapsular lesion, Articular knee cartilage

## Abstract

**Background:**

MRI is the most accurate imaging modality for diagnosing knee pathologies. However, there is uncertainty concerning factors predicting false negative MRI, such as meniscal tear patterns as well as patient factors. The aims of this study were to report 1.5-Tesla MRI accuracy of ACL, meniscus and articular cartilage damage and characterize false negative lesions.

**Methods:**

Two hundred eighteen consecutive knee arthroscopies performed in our institution between 2013 and 2016 and their respective prospectively-collected MRI reports were reviewed. Inclusion criteria were age > 15 years-old, primary arthroscopy, 1.5-Tesla MRI performed at the same institution, and time interval MRI-surgery < 6 months. Exclusion criteria were revision arthroscopy and arthroscopic-assisted fracture fixation or multiligament surgery. Accuracy measures and Kappa coefficients were calculated comparing the MRI diagnosis to the arthroscopic findings. Moreover, the arthroscopic findings of false negative MRI were compared to the findings of true positive MRI using the Fisher-exact test. Pearson correlation was used for testing the correlation between MRI accuracy and patient age.

**Results:**

The highest accuracy was observed in medial meniscus and in ACL findings. For the medial meniscus sensitivity, specificity, agreement, and Kappa coefficient were 77, 92, 86%, and 0.7, and for the ACL these measures were 82, 97, 87%, and 0.73. MRI accuracy was lower in the lateral meniscus and articular cartilage with Kappa coefficient 0.42 and 0.3, respectively. More specifically, short peripheral tears in the posterior horn of the medial meniscus were characteristic of false negative findings compared to true positive findings of the MRI (*p* <  0.01). MRI accuracy correlated negatively compared to arthroscopic findings with patient age for the medial meniscus (*r* = − 0.21, *p* = 0.002) and for articular cartilage damage (*r* = − 0.45, *p* <  0.001).

**Conclusion:**

1.5-Tesla MRI will accurately diagnose ACL and medial meniscal tears and can reliably complete the diagnostic workup following physical examination, particularly in young adults. This modality however is not reliable for diagnosing short peripheral tears at the posterior horn of the medial meniscus and partial thickness articular cartilage lesion of the femoral condyles. For these lesions, definitive diagnosis may require cartilage-specific MRI sequences or direct arthroscopic evaluation.

**Level of evidence:**

Prognostic study, Level III.

## Background

Arthroscopy is considered the “gold-standard” for the diagnosis of internal knee pathologies aside of being a minimal invasive surgical procedure to treat intraarticular lesions [1]. Yet, arthroscopic intervention has potential complications, some of which could be lethal such as pulmonary embolism [2], and therefore its use should be implemented cautiously and guided according to appropriate indications. Magnetic Resonance Imaging (MRI), although questioned almost three decades ago concerning clinical value and cost-effectiveness for knee disorders [3], has gained popularity as the best noninvasive diagnostic modality and is currently widely used for the evaluation of intra-articular knee lesions [4]. Along the years, this imaging modality was the focus for studies that explored its diagnostic accuracy compared to knee arthroscopy as the “gold-standard”. The anterior cruciate ligament (ACL) and the menisci were the most commonly investigated structures in this respect. Accuracy measures of MRI for these structures range from 80 to 95% in most studies [5–12]. Contrary, accuracy of MRI for knee articular cartilage lesions is more controversial with several reports showing sensitivity as low as 15% and as high as 60% depending on the depth of the lesion [13–15]. Only few studies were designed to identify factors associated with false negative MRI. Several researchers identified specific tear patterns of the menisci that were more likely to present false negative on MRI. The lateral meniscus showed decreased MRI sensitivity for peripheral longitudinal tears at the posterior horn [8, 16]. Another study showed similar tear characteristics in the medial meniscus that were associated with false negative MRI (i.e. peripheral longitudinal tears at the posterior horn) when these occurred concomitantly with ACL tears [17]. A short time interval from the injury to performing the MRI was also associated with false negative MRI for meniscal tears but only in the case of concomitant ACL tears [17] and not when the meniscal tear was isolated without accompanying ACL damage [8]. Awareness of the aforementioned information concerning specific factors which were found to be associated with a false negative MRI could potentially assist in improving MRI interpretation, although there were a few limitations of previously reported data on MRI accuracy that should be remembered. These include incorporating low-magnet strength MRI which is rarely used today for the diagnosis of knee lesions (i.e. lower than 1.5-Tesla) [1], not indicating accuracy measures for specific areas in the meniscus in some studies (i.e. anterior horn, body, posterior horn) [1, 10, 11], and using multiple MRI sequencing techniques in a single series of patients [1]. Because MRI accuracy depends on magnet strength [1, 10], on the specific location of the lesion, and possibly on aging changes that occur within these intraarticular structures [18–20], more specific data about MRI accuracy could be useful to improve clinical judgement when viewing the MRI as an important step during management and decision making for a suspected knee lesion. In addition, with the increasing surgeons’ awareness of subtle lesions in recent years that might be challenging to identify on MRI it is possible that MRI accuracy measures would evolve over time. Such subtle lesions include small medial meniscocapsular lesions [21], lateral meniscocapsular (i.e. popliteo-meniscal fasciculi) lesions that result in meniscus hypermobility [22, 23] and medial meniscus ramp lesions which are challenging to identify even during arthroscopy unless observed from a posteromedial view through the intercondylar notch or through a separate posteromedial portal [24]. In fact, a large systematic review also demonstrated a trend for negative correlation between reported accuracy of MRI for meniscal lesions and the year of publication [7]. The purpose of the current study was therefore to add to the body of literature on current accuracy measures specific to 1.5-Tesla MRI of the knee in the adult population relating to the ACL, the menisci and to the articular cartilage and to identify characteristics of false negative lesions. It was hypothesized that MRI accuracy would be between 80 to 90% for the menisci and ACL and substantially lower for the articular cartilage. It was also hypothesized that specific lesion characteristics of the menisci and articular cartilage in addition to patient age would be associated with MRI accuracy.

## Methods

Operative room registry was reviewed to identify all knee arthroscopies performed in our institution between 2013 and 2016 and their respective preoperative prospectively-collected MRI reports. The status of the ACL, the menisci and the articular cartilage was collected from the surgical reports and from their respective preoperative MRI reports by investigators which were not involved in the surgeries or in the MRI evaluations (JEJK, RBE, BK). Inclusion criteria were: (1) patient age older than 15 years, (2) primary knee arthroscopy, (3) MRI performed at the same institution (MOR-MAR Ltd.), and (4) time interval between the MRI scan and surgery shorter than 6 months. Exclusion criteria were: (1) revision knee arthroscopy, (2) arthroscopic-assisted fracture reduction surgery (i.e. repair of ACL avulsion fracture of the tibial eminence), and (3) multiligament knee surgery. The MRI used a 1.5-Tesla magnet and the standard protocol included the Turbo Spin Echo (TSE) technique including proton density (PD), T2- and T1-weighted sequences with fat suppression and Short Tau Inversion Recovery (STIR) sequences with sagittal, coronal and axial cuts. The interpretation of the preoperative MRI scans was performed by a musculoskeletal imaging specialized radiologist. The MRI criteria used to define lesions of the menisci and ACL were in accordance with previous descriptions. The presence of an intra-meniscal signal extending to an articular surface and/or distortion of the normal meniscus shape represented on the MRI a clinically meaningful tear [25]. Signs of a complete ACL tear included discontinuity of the ACL fibers, wavy appearance, and an angle of less than 45° between the distal ACL fibers and the tibia [4]. In addition, secondary signs of an ACL tear included bone bruise at the anterior or central lateral femoral condyle and posterolateral tibial plateau with or without countercoup bone contusion at the posteromedial tibial plateau [4]. The Segond Fracture was also considered a sign of a complete ACL tear [4]. Partial ACL tears were defined when these signs were more subtle, but since most traumatic partial ACL tears are associated with a positive pivot shift and thus representing a clinically meaningful ACL tear both partial and complete ACL tears were considered as positive ACL tears in accordance with previous investigations on the accuracy of MRI [11].

### Statistical analysis

Sample size for this study was planned to be in accordance with a recent systematic review and meta-analysis of the diagnostic accuracy of MRI for suspected ACL and meniscal tears of the knee [11]. This meta-analysis included studies that focused on adult population using 1.5-Tesla MRI which are similar characteristics to our study. The smallest series in that meta-analysis included 23 patients and the largest 244 patients [11]. Percent of agreement and Kappa coefficients were calculated comparing the MRI radiologist’s interpretations and the arthroscopic findings for each intraarticular structure (i.e. the meniscus, the articular cartilage and the ACL). Kappa coefficient values for the MRI accuracy were interpreted as “almost perfect” for the range 0.81–1.00, “substantial” for the range 0.61–0.80, “moderate” for the range 0.41–0.60, “fair” for the range 0.21–0.40, and “slight” for the range 0.00–0.20. In addition, for each tested area, sensitivity {(True Positive) / (True Positive + False Negative) * 100} and specificity {(True Negative) / (True Negative + False Positive) * 100} were calculated. In order to test whether specific tear morphologies characterized false negative MRI as opposed to true positive MRI, the arthroscopic findings of false negative MRI cases were compared to the arthroscopic findings of true positive MRI lesions in each area of the meniscus and the articular cartilage. The Fisher-exact test was used to compare lesion characteristics between the false negative and true positive MRI groups. Pearson correlation coefficients were calculated for testing the correlation between the level of agreement over all areas (i.e. ACL, menisci, and articular cartilage) and age of the patient. Since there were several musculoskeletal radiologists involved in interpreting the MRIs, Pearson correlation coefficients were also calculated between the level of agreement over all parameters and the radiologist number of years of experience. This correlation analysis was also repeated for the level of agreement over medial meniscus and lateral meniscus areas exclusively. All tests were two-tailed, and a *p*-value of 5% or less was considered statistically significant. The data was analysed using the SAS® version 9.3 (SAS Institute, Cary, North Carolina).

## Results

Inclusion criteria were fulfilled by 218 cases. Patient median age was 35 (Q1 = 23, Q3 = 49, range = 15–76) years. There were 168 males (77.4%). Percent of agreement between MRI findings and arthroscopic findings over all areas and all patients was 90.4%.

Table 1 presents sensitivity, specificity, and percent of agreement and Kappa coefficients for each tested area. Table 2 presents comparisons of lesion characteristics between false negative and true positive MRI for the meniscus and articular cartilage.

**Table 1:** Sensitivity, specificity, percent of agreement and Kappa coefficients for each area

Tested area	Sensitivity [%]	Specificity [%]	Agreement [%]	Kappa coefficient
**MM (all areas)**	77	92	86	0.70
MM AH	88	47	84	0.27
MM Body	72	60	69	0.27
MM PH	77	91	85	0.69
**LM (all areas)**	83	57	73	0.42
LM AH	83	19	78	0.01
LM Body	91	41	83	0.34
LM PH	86	48	76	0.36
LM Discoid	99	17	97	0.21
**ACL**	82	97	87	0.73
**Articular Cartilage (all areas)**	81	49	65	0.30
Medial femoral condyle	94	32	74	0.30
Lateral femoral condyle	93	22	87	0.14
Medial tibial condyle	90	39	83	0.30
Lateral tibial condyle	95	8	79	0.04
Patella	95	47	87	0.48
Trochlea	99	9	85	0.12

*MM* Medial Meniscus, *AH* Anterior Horn, *PH* Posterior Horn, *LM* Lateral Meniscus

**Table 2:** Comparisons of lesion characteristics between false negative and true positive MRI for the meniscus and articular cartilage

Lesion	FN MRI group	TP MRI group	*P* value
**Medial meniscus tear**	***n*** **= 7**	***n*** **= 122**	
^*^Short peripheral tear of posterior horn	*n* = 7	*n* = 1	< 0.01
Root tear	*n* = 0	*n* = 7	ns
Flap/ vertical oblique/ complex tear	*n* = 0	*n* = 63	0.01
Bucket handle tear	*n* = 0	*n* = 27	ns
Radial tear	*n* = 0	*n* = 7	ns
Horizontal split and other degenerative tears	*n* = 0	*n* = 17	ns
Concomitant ACL tear	*n* = 5	*n* = 24	< 0.01
**Lateral meniscus tear**	***n*** **= 32**	***n*** **= 47**	
^*^Short peripheral tear of posterior horn	*n* = 3	*n* = 1	ns
Posterior horn partial thickness stable tear	*n* = 18	*n* = 4	< 0.01
Full thickness longitudinal or flap tear	*n* = 5	*n* = 30	< 0.01
Bucket handle tear	*n* = 0	*n* = 3	ns
Radial tear with or without horizontal split	*n* = 5	*n* = 9	ns
Root tear (underwent repair)	*n* = 1	*n* = 0	ns
Concomitant ACL tear	*n* = 20	*n* = 22	ns
**Cartilage lesion (all areas)**	***n*** **= 56**	***n*** **= 53**	
Grade [1 or 2] vs. Grade [3 or 4] on arthroscopy	*n* = 41 vs. 15	*n* = 21 vs. 32	< 0.01
**Cartilage lesion Grade [3 or 4] on arthroscopy**	***n*** **= 15**	***n*** **= 32**	
Femoral condyle lesions	*n* = 12	*n* = 26	ns
Tibial condyle lesions	*n* = 2	*n* = 14	ns
Patella lesions	*n* = 2	*n* = 9	ns
Trochlea lesions	*n* = 4	*n* = 13	ns

*FN* False Negative, *TP* True Positive, *ns* Non-significant; ^*^, This lesion induced instability of the posterior horn and was repaired using 1 or 2 sutures. For the medial meniscus, this lesion is also termed “Ramp lesion” or “Small medial meniscocpasular lesion”

**Fig. 1:**
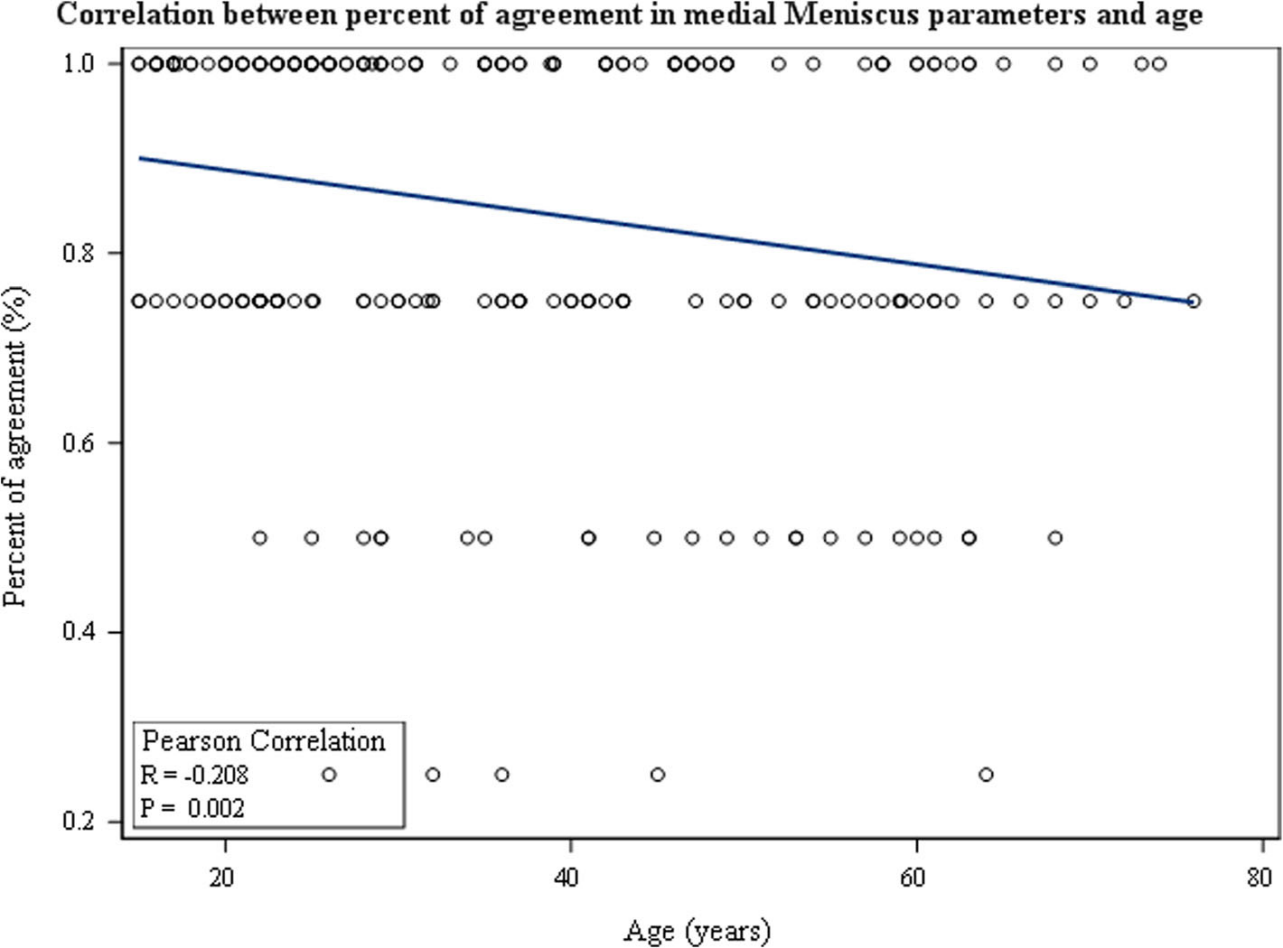
Pearson correlation between percent of agreement in medial meniscus parameters and age of the patients

**Fig. 2:**
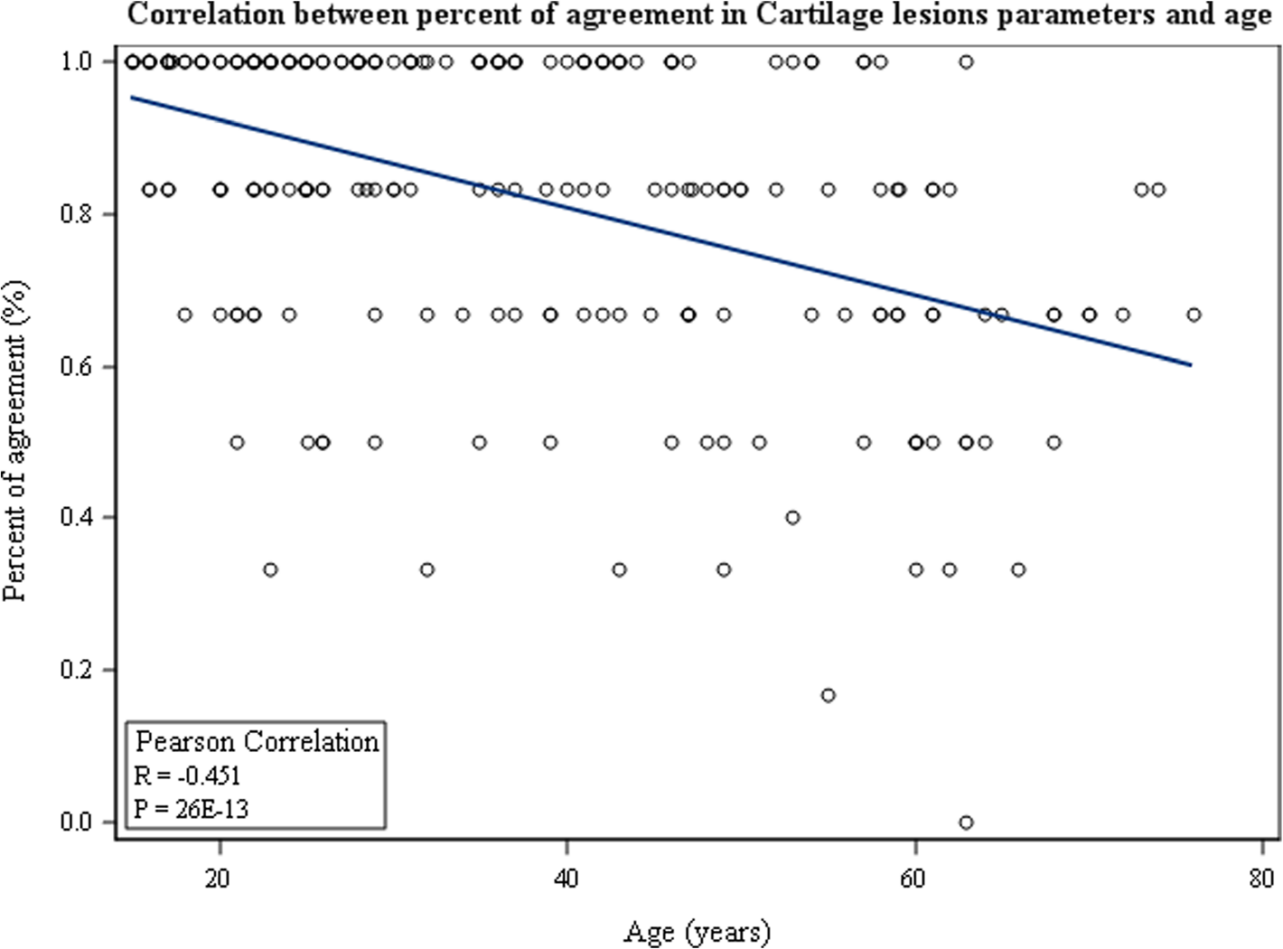
Pearson correlation between percent of agreement in cartilage lesions parameters and age of the patients

Regarding the correlation between patient age and MRI accuracy, it was demonstrated that patient age had negative impact on MRI accuracy for medial meniscus tears (r = − 0.21, *p* = 0.002) (Fig. 1) and for articular cartilage lesions (r = − 0.45, *p* <  0.001) (Fig. 2). No significant correlation was demonstrated between patient age and MRI accuracy regarding lateral meniscus tears (*p* = 0.3) or ACL tears (*p* = 0.3).

There were 13 musculoskeletal radiologists involved in interpreting all 218 cases. Each case had one MRI report corresponding to one radiologist. Number of years of experience in musculoskeletal radiology among the 13 radiologists ranged 3 to 24 years (mean, 12 ± 6). No statistically significant correlation was found between percent of agreement over all areas and the radiologist number of years of experience. Looking at the specific examined areas, a positive correlation was found in this regard only for the lateral meniscus and this correlation accounted for only 2% of the variance (r = 0.16, r^2^ = 0.02, *p* = 0.01).

## Discussion

The following five key findings summarize this study which investigated current accuracy measures of 1.5-Tesla knee MRI: (1) ACL lesions are reliably diagnosed on 1.5-Tesla MRI with specificity reaching nearly 100% and sensitivity over 80%. (2) Medial meniscal lesions at the posterior horn have overall similarly high MRI sensitivity and specificity as ACL lesions, with the exception of short peripheral or meniscocapsular tears as a specific tear pattern at this area which should be diagnosed definitively only by direct arthroscopic evaluation. (3) Lateral meniscus lesions have high sensitivity similarly to medial meniscal lesions, but low specificity and only “fair” Kappa coefficients. This implies overall reduced MRI accuracy for this meniscus with higher false positive MRI diagnosis of meniscal lesions which may not be confirmed by the arthroscopic evaluation and this is particularly noticeable for the anterior horn area. (4) Articular cartilage lesions have high sensitivity but low and variable MRI specificity and only “fair” Kappa coefficients for most knee compartments. (5) MRI accuracy for medial meniscus tears and articular cartilage lesions decreases with patient age.

For ACL and medial meniscal posterior horn lesions in general, this study demonstrated the highest accuracy among the intraarticular areas tested. 1.5-Tesla MRI can therefore be considered a highly reliable modality for diagnostic purposes in these areas. This is in accordance to previous reports [5–12] but should be still interpreted with caution since Kappa coefficients in these areas was “substantial” and not “perfect”. The medical history and physical examination should therefore play a role even for these “MRI-reliable” areas during the diagnostic process whereas arthroscopic evaluation still remains the “gold-standard” diagnostic modality. The anterior horn area of both menisci showed in our study only “fair” and “slight” MRI accuracy with the lowest specificity of all meniscal areas (i.e., anterior horn, body, posterior horn). This area should therefore be viewed as less consistent for diagnostic purposes on MRI. Of note, the anterior horns can be challenging to fully appreciate and probe during arthroscopic inspection when using standard anterolateral and anteromedial portals and a 30° arthroscope as was used in this series and this could affect the reliability of the arthroscopic inspection used as the “gold-standard” diagnostic tool. Further observations may be considered using a 70° arthroscope or creating additional portals in order to learn if this would provide a better appreciation of the anterior horns of the menisci and potentially change to diagnosis of lesions in these areas.

With regard to the posterior horn of the medial meniscus, short peripheral lesions were “blind spots” for MRI diagnostic purposes. These subtle lesions were reported as clinically meaningful lesions that should be identified and repaired arthroscopically [21, 24]. In this series, these short peripheral tears were repaired by using 1or 2 sutures to firmly stabilize the posterior horn of the meniscus. It should be remembered however that these short subtle peripheral lesions termed sometimes “Ramp lesions” were also reported as a diagnostic challenge during arthroscopic inspection [24]. This lesion is practically a meniscocapsular lesion involving the meniscocapsular junction or the very peripheral rim of the medial meniscus which is located at the posterior horn in proximity to the meniscal root. Increased mobility of the posterior horn of the meniscus in these cases can be observed by arthroscopic probing and should raise the suspicion of such a hidden lesion. However, in order to clearly identify these lesions, a posteromedial view may be necessary which could be executed by either passing the arthroscope through the intercondylar notch or by creating a separate posteromedial portal [24]. In rare cases a short medial meniscocapsular lesion which was not identified on the preoperative MRI could be identified arthroscopically without the presence of an accompanying ACL lesion and should be repaired in order to stabilize the posterior horn of the meniscus [21]. Regarding false negative MRI lesions of the lateral meniscus, these mostly appeared as partial thickness, stable tears, of the posterior horn. These lesions were “left alone” during the arthroscopic management of these patients due to their good prognosis [26]. The clinical significance of false negative MRI in this specific subtype of a meniscal tear is therefore questionable.

Concerning articular cartilage lesions, 1.5-Tesla MRI had in this study an overall low accuracy compared to other knee structures tested. The grade of the lesion more than the location of the lesion, was found to be associated with false negative MRI findings, with severity grades 1 and 2 (i.e. partial thickness lesions) often not diagnosed by the standard 1.5-Tesla MRI. Of the knee compartments, the patella had the highest Kappa coefficient for MRI accuracy which was interpreted as “moderate”. Therefore, considering the overall relatively low reliability of diagnosing low grade articular cartilage lesions throughout the knee, the patella may be considered an acceptable area for using MRI diagnosis. This is true particularly for lesions which are nearly full-thickness (i.e. grades 3 or 4). Such lesions may be severe enough to cause symptoms and be clinically relevant. Other investigators have previously mentioned the low sensitivity of MRI in diagnosis of partial-thickness articular cartilage lesions [13–15]. However, it was also demonstrated that although conventional MRI planes could not always clearly visualize the articular cartilage lesions, particularly of the posterior aspect of the femoral condyles because of the convexity of the condyles, MRI planes which were obtained perpendicular to the injured surface improved dramatically the visualization of the lesions [13]. Accordingly, it is possible that since the patella is characterized not only as having the thickest cartilage among the knee compartments but as also having a more flat chondral surface, which is not as convex as the surface of the femoral condyles, the standard axial MRI plane which is almost perpendicular to the patella chondral surface, may have shown in this study increased accuracy for diagnosing articular cartilage lesions of the patellofemoral joint as compared to lesions of the femoral condyles. Advanced articular cartilage MRI imaging modalities could provide improved diagnostic ability when compared to the standard 1.5-Tesla MRI used in this study, in respect to the articular cartilage lesions. The improved MRI modalities could identify compositional changes at the molecular level of the articular cartilage at an early stage of cartilage degeneration. These include T2 mapping, delayed gadolinium enhanced magnetic resonance imaging of cartilage (dGEMRIC), T1 rho, and others [27–29]. These advanced techniques, however, require longer acquisition times compared to conventional 2D MRI and are therefore less feasible for most conventional MRI facilities. Overall, this study emphasizes the limitations of 1.5-Tesla MRI for diagnosis of articular knee cartilage lesions and particularly for partial thickness lesions over the condyles.

MRI accuracy regarding medial meniscus tears and articular cartilage lesions decreased with patient age. This may be related to the compositional changes occurring within these tissues such as increased cross-linking of collagen and calcium deposits in the meniscus, and reduced water content and proteoglycans and decreased matrix synthesis in the articular cartilage [18, 20]. These aging changes result in greater susceptibility of these tissues to microtrauma. This in turn may result in further difficulty in interpreting the MRI signals as “aging per se” as opposed to a “true tear”. In older populations, therefore, reduced diagnostic accuracy of 1.5-Tesla MRI should be taken into consideration during the diagnostic process.

Concerning the aforementioned meniscal and articular knee cartilage lesions which were false negative in our study on the standard 1.5-Tesla MRI it is worth being aware of the technique of in-office needle arthroscopy systems, which have been introduced and studied in relation to their accuracy in diagnosing intraarticular knee pathologies [30, 31]. While these systems do not possess yet complete capabilities to diagnose and treat subtle lesions as standard arthroscopic systems do, their accuracy seems superior to MRI for diagnostic purposes in some knee lesions. Such incisionless minimally invasive systems may be one option to bridge the diagnostic gap between the two modalities without increasing the procedural risks. In addition, in this study, only 1.5-Tesla magnet MRI was used. This is of value because this magnet is considered the backbone of knee MRI evaluation [4]. However, since MRI magnetic field strength also plays a role in the accuracy of MRI diagnoses [1, 10], the findings presented can be applicable to most standard imaging facilities but should not be extrapolated to MRIs with stronger magnets which may show greater accuracy.

Limitations of this study include the retrospective design and the variations in radiologists’ interpretations of knee MRI without calculating inter- and intra-observer agreement. Moreover, because the number of MRI cases was not equally distributed among the radiologists in this study as a result of the retrospective design, variation in radiologists’ interpretation of the MRIs for different knee lesions was reported in relation to radiologists’ years of experience. This was performed despite the fact that years of experience may not be the only factor potentially affecting MRI interpretation among different radiologists. While accepted MRI criteria for meniscal tears, ACL tears, and articular cartilage lesions were used [4, 11, 25], MRI signals may have still been subjectively interpreted by the different observers. Despite these limitations, the Coleman methodology score of this study calculated 60 points. This is higher than the mean Coleman score of 54 points achieved by other studies which were discussed in a large systematic review addressing basically similar research questions [7]. This is supportive of that the methodology of this study is “acceptable” although not “perfect” and still superior to most other studies in the literature which were discussed in that review [7].

## Conclusion

1.5-Tesla MRI will accurately diagnose ACL and medial meniscal tears and can reliably complete the diagnostic workup following physical examination, particularly in young adults. This modality however is not reliable for diagnosing short peripheral tears at the posterior horn of the medial meniscus and partial thickness articular cartilage lesion of the femoral condyles. For these lesions, definitive diagnosis may require cartilage-specific MRI sequences or direct arthroscopic evaluation.

## Data Availability

The datasets used during this study will be available from the corresponding author, i.e. Iftach Hetsroni, on request.

## Abbreviations

MRI: Magnetic Resonance Imaging
ACL: Anterior Cruciate Ligament;
MM: Medial Meniscus
AH: Anterior Horn
PH: Posterior Horn
LM: Lateral Meniscus
FN: False Negative
TP: True Positive
ns: Non-significant
